# Supplementary material for: CD11b Deficiency Favors Cartilage Calcification via Increased Matrix Vesicles, Apoptosis, and Lysyl Oxidase Activity
Source: Int J Mol Sci. 2023 Jun 5;24(11):9776. doi: 10.3390/ijms24119776 (PMC10253935; doi:10.3390/ijms24119776)
Supplement: Supplementary file 1 [file ijms-24-09776-s001.zip › ijms-2369388-supplementary.pdf]

International Journal of *Molecular Sciences*

MDPI

GEO	Date	Author	Database main focus	Model and number of patients/animals	CD11b gene expression
**GSE114007**	2018	Fisch KM et al.	Identification of transcription factors responsible for dysregulated networks in human osteoarthritis cartilage by global gene expression analysis	Human knee cartilages<br>20 OA vs. 18 normal	Yes
**GSE117999**	2018	Rai MF et al.	Transcriptome comparison of cartilage from patients with and without osteoarthritis	Human knee cartilage<br>12 OA vs. 12 non-OA	Yes
**GSE104782**	2018	Ji Q et al.	Single-cell RNA-seq analysis reveals the progression of human osteoarthritis	Human knee chondrocytes<br>10 OA	Yes
**GSE151937**	2020	Datta P et al.	mRNA sequencing of human osteoarthritis chondrocytes treated with or without autotaxin inhibitor	Human knee chondrocytes<br>4 OA	Yes
**GSE171952**	2021	Mimpen JY et al.	Interleukin-17A causes osteoarthritis-like transcriptional changes in human osteoarthritis-derived chondrocytes and synovial fibroblasts in vitro	Human knee chondrocytes<br>24 OA	Yes
**GSE172291**	2021	Liang T et al.	Inhibition of nuclear receptor RORα attenuates cartilage damage in osteoarthritis by modulating IL-6/STAT3 pathwa	Human knee chondrocytes<br>8 OA vs 8 non-OA	No
**GSE85254**	2017	Weinmann D et al.	Galectin-3 Induces a Pro-degradative/inflammatory Gene Signature in Human Chondrocytes, Teaming Up with Galectin-1 in Osteoarthritis Pathogenesis	Human knee chondrocytes<br>4 OA	Yes
**GSE150411**	2020	Reed KS et al.	Transcriptional response of fibronectin fragment treatment in human articular chondrocytes	Human ankle chondrocytes<br>3 OA	Yes
**GSE111357**	2019	Young DA et al.	Osteoarthritis RNA Sequencing from ground cartilage samples	Human hip cartilage<br>10 OA vs 6 non-OA	Yes
**GSE57218**	2014	Ramos YF et al.	Gene expression profiles from joint-matched macroscopically intact and OA affected cartilage of patients undergoing joint replacement surgery due to end-stage OA	Human knee and hip cartilage<br>33 OA	Yes

Table S1. Analysis of open access GEO databases on human and murine cartilage gene expression. Expression of ITGAM was assessed by number of reads (> 10).

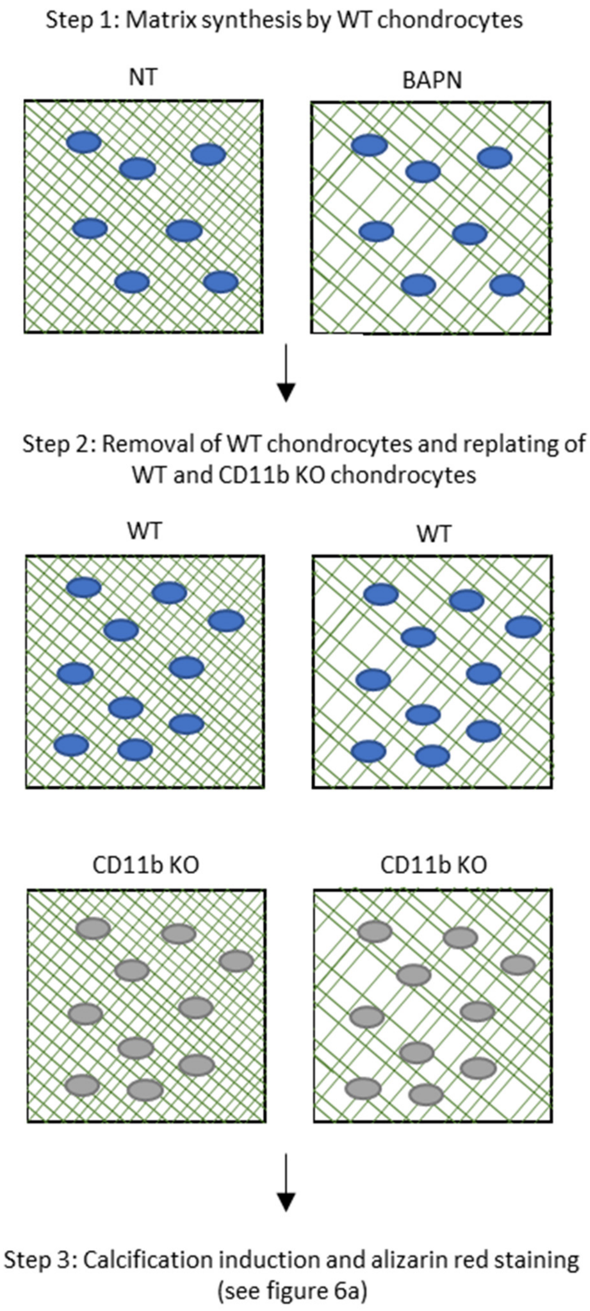

Figure S1. Two-steps experiment graphical explanation.
